# Time for a Systems Biological Approach to Cognitive Aging?—A Critical Review

**DOI:** 10.3389/fnagi.2020.00114

**Published:** 2020-05-12

**Authors:** Deena Ebaid, Sheila G. Crewther

**Affiliations:** Department of Psychology and Counselling, School of Psychology and Public Health, College of Science, Health and Engineering, La Trobe University, Melbourne, VIC, Australia

**Keywords:** cognitive aging, cognitive decline, theories of aging, biological aging, cardiovascular health, systems neuroscience

## Abstract

The underlying premise of current theories of cognitive decline with age tend to be primarily cognitive or biological explanations, with relatively few theories adequately integrating both aspects. Though literature has also emphasized the importance of several factors that contribute to cognitive aging including: (a) decline in sensory abilities; (b) the effect of motor speed on paper-pencil measures of cognitive speed; (c) the impact of level of education and physical activity; and (d) molecular biological changes that occur with age, these factors have seldom been implicated into any single theoretical model of cognitive aging. Indeed, such an integrated bio-cognitive model of aging has the potential to provide a more comprehensive understanding of attention, perception, learning, and memory across the lifespan. Thus, the aim of this review was to critically evaluate common theories of age-related cognitive decline and highlight the need for a more comprehensive systems neuroscience approach to cognitive aging.

## Introduction

Many theories of cognitive aging have been proposed to account for the declines observed in cognitive performance across the healthy lifespan, where slowing of processing speed is one of the most common markers of cognitive aging (Salthouse, 1996; Ebaid et al., 2017; Brown et al., 2019). Some explanations are predominantly cognitive, while others have described a more biological basis to account for the decline in cognitive performance (Baltes and Lindenberger, 1997). To date however, no single explanation has incorporated all these aspects of biological change to adequately and appropriately account for the decline in cognitive processing seen in healthy aging. With the increase of life expectancy, understanding the changes that would be expected to affect cognitive processing during a healthy lifespan has become a public health and socioeconomic priority. Thus, this review will critically evaluate prominent theories of cognitive aging, which are primarily based on the frameworks of cognitive psychology, and then highlight the need for a newer more comprehensive systems neuroscience approach to cognitive aging (Table 1 provides a summary of theories discussed in this review). The biological changes with age discussed in this review were chosen based on their well-documented relationship with age-related cognitive decline, and include hypertension (De Silva and Miller, 2016), hyperlipidemia (Cheng et al., 2014), obesity (Dahl and Hassing, 2013) and anxiety (Gulpers et al., 2016). These factors were also chosen due to their associated risk for cardiovascular disease which is the leading cause of mortality worldwide (World Health Organisation, 2019). Though several additional biological changes with age exist and have been well-documented in the aging literature, the current review will only focus on a select few, to discuss them in the context of cognitive aging. Thus, some factors examined in this review only apply to a subset of older adults who suffer from the particular health condition being discussed.

**Table 1:** Summary of theories pertaining to age-related cognitive decline.

Theory/Hypothesis	Reference/s	Summary	General limitations
The Common-Cause Hypothesis	Baltes and Lindenberger (1997)	• A common biological factor can account for the age-related variance in sensory, sensorimotor and intellectual functioning.	• The focus on one common biological factor neglects much evidence and constructs associated with comprehensive network-based approach to cognitive functioning.
		• Dopaminergic functioning has been proposed as that “common factor” (Li and Lindenberger, 1999).	• Does not consider the role of other monoamines such as noradrenaline and serotonin that modulate neural network activation and neglects other age-related molecular changes.
The Sensory Deprivation Hypothesis	Oster (1976) and Valentijn et al. (2005)	• A lack of adequate sensory input over a prolonged period results in neuronal atrophy, in turn impairing cognitive function.	• Localises cognitive decline to the neural networks dedicated to visual and auditory processing.
			• Neglects a comprehensive neural network-based approach to cognitive functioning.
The Information Degradation Hypothesis	Schneider and Pichora-Fuller (2000)	• Perceptual signals are weakened or *degraded* due to age-related impairments, resulting in impaired cognitive processing.	• Age-related differences on cognitive tasks are still demonstrated when sensory deficits (in vision and hearing) are corrected (Hall et al., 2005; Anstey et al., 2006).
			• Neglects a comprehensive neural network-based approach to cognitive functioning.
The Processing Speed Theory of Adult Age Differences in Cognition	Salthouse (1996)	• A reduction in the speed at which cognitive operations can be executed, underlies the decline observed in more complex cognitive functions, including memory, problem solving and reasoning.	• Based on findings from paper-pencil measures used with aging populations, without consideration of the confound of motor impairment with age.
The Inhibitory Deficit Hypothesis	Hasher and Zacks (1988)	• An attention-based model which suggests that good cognitive task performance requires efficient processing of relevant information while simultaneously inhibiting irrelevant information.	• Attention and inhibition of stimuli are highly variable and dependent on the cognitive task (Rey-Mermet and Gade, 2018).
		• Age-group differences in task performance are due to older adults being less efficient in selectively attending to task-relevant stimuli while simultaneously inhibiting task-irrelevant information.	• Tasks used to measure inhibitory control often rely on motor accuracy and response speed on a computerised task, or verbal reaction times, which introduce the potential confound of hand motor speed or slower orofacial movement in an aged population.
The Scaffolding Theory of Aging and Cognition (STAC)	Park and Reuter-Lorenz (2009)	• The STAC integrates evidence from structural and functional neuroimaging to provide a conceptual model of cognitive aging.	• Unclear exactly how compensatory or scaffolding mechanisms are utilized, and how much these compensatory pathways actually contribute to “better” performance.
		• The model proposes that the level of cognitive functioning that an older individual achieves is a consequence of both neural/functional deterioration that requires and utilises compensatory ‘scaffolding’ to attenuate the adverse effects of the neural and functional decline.	• From the viewpoint of STAC, it is unclear how factors that are considered protective against cognitive aging such as higher levels of education or physical activity, strengthen the “scaffolding” mechanism.
The Cognitive Reserve Hypothesis	Stern (2002)	• This hypothesis postulates that individuals who possess a greater ability to recruit and utilise more brain regions, are better able to cope with a greater level of age-related brain pathology before clinical diagnosis is reached.	• Methods of quantifying “cognitive reserve” typically involve inferring reserve from proxy measures such as educational attainment or current occupation. These vary from study to study.
		• It is suggested that this is enabled by means of neural compensation and recruitment of additional brain regions that lead to intact behavioural performance on cognitive tasks.
The Frontal Aging Hypothesis	Jackson (1958) and Dempster (1992)	• This hypothesis is based on the notion that selective frontal lobe pathology in the form of reduced volume, metabolism and simultaneous decline in grey and white matter integrity underlie the cognitive deficits observed in healthy aging.	• Older adults are also impaired in cognitive abilities that are often largely independent of prefrontal areas (Greenwood, 2000).
		• The frontal lobe pathology has predominantly been demonstrated using function magnetic resonance imaging fMRI or PET.	• Whole brain imaging shows that other cortical areas including the temporal and parietal lobes also show compromised integrity with age.
			• Neglects a comprehensive neural network-based approach to cognitive functioning.
The Hemispheric Asymmetry Reduction in Older Age (HAROLD) model	Cabeza (2002)	• The HAROLD model is based on the premise that age is related to decreases in lateralisation of brain function, which stem from fMRI observations that young and older adults recruit different neural networks during the same cognitive task (particularly episodic and working memory tasks).	• The HAROLD model has been exclusively described for the PFC, and insight into this model has come from studies using mainly episodic and working memory tasks.
		• It has been reported that young adults display left PFC activation during verbal working memory tasks and right PFC activation during spatial working memory tasks, whereas older adults demonstrate bilateral activation of the PFC while engaging in both verbal and spatial working memory tasks (Reuter-Lorenz et al., 2000).	• Localises cognitive decline to a specific brain region.
			• Neglects a comprehensive neural network-based approach to cognitive functioning.
The Compensation-Related Utilization of Neural Circuits Hypothesis (CRUNCH)	Reuter-Lorenz and Cappell (2008)	• The CRUNCH model suggests that declines in neural efficiency across the lifespan results in older adults recruiting more neural resources predominantly in the dorsolateral prefrontal cortex compared to young adults when task demands are low.	• Localises cognitive decline to a particular brain region.
		• However, as task demands increase, neural activation for younger adults exceeds that of older adults, and task performance for older adults is also impaired.	• Neglects a comprehensive neural network-based approach to cognitive functioning.
		• When task demands exceed a certain level of difficulty, the aging-brain “falls short” of sufficient activation levels, and task performance declines compared to the younger adults.

## Sensory System Decline

### The Sensory Deprivation Hypothesis, the Information Degradation Hypothesis, and the Common-Cause Hypothesis

**Figure 1:**
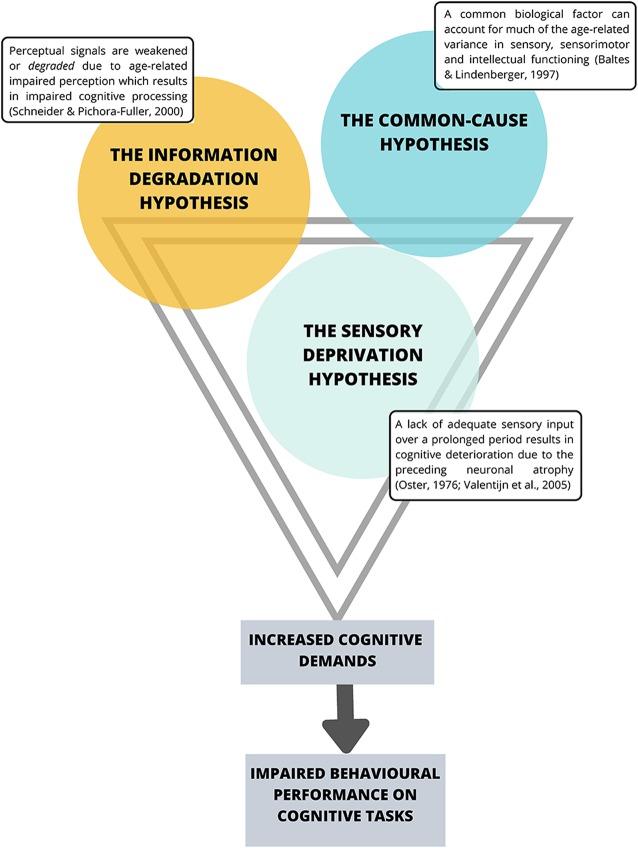
Current theories suggesting an interaction between sensory system decline and impaired cognitive performance. Note that the premises behind these theories share substantial overlap.

The *Sensory Deprivation Hypothesis*, the *Information Degradation Hypothesis*, and the *Common-Cause Hypothesis* collectively suggest a strong interaction between declines in the visual and auditory sensory systems and a decline in cognitive performance (see Figure 1).

The *Sensory Deprivation Hypothesis* suggests that a lack of adequate sensory input over a prolonged period is likely to result in cognitive deterioration due to the preceding neuronal atrophy (Oster, 1976; Valentijn et al., 2005). Similarly, the *Information Degradation Hypothesis* states that when perceptual signals are weakened or *degraded*, either due to experimental manipulations or age-related impaired perception, higher-order cognitive processes are in turn affected (Schneider and Pichora-Fuller, 2000) presumably because the cognitive load is greater for weak perceptual signals, and thus requires more cognitive resources to interpret the signal, which compromises cognitive performance (Zekveld et al., 2011). *The Common-Cause Hypothesis* (Baltes and Lindenberger, 1997) suggests concurrent peripheral and central decline occurring simultaneously with declines in aspects of conscious cognition, and proposes that sensory and cognitive function are both likely to be an expression of the *“physiological architecture of the aging brain”* (p. 13). More specifically, the premise of this hypothesis is that as age increases, a common biological factor can account for much of the age-related variance in sensory, sensorimotor and intellectual functioning, where dopaminergic functioning, has been proposed as that common factor (Li and Lindenberger, 1999). In particular, Li and Lindenberger (1999) have suggested that age-related differences in pre-synaptic markers such as the binding potential for the dopamine (DA) transporter (Erixon-Lindroth et al., 2005) and post-synaptic markers such as D_1_receptor densities (Wang et al., 1998) and D_2_ (Bäckman et al., 2000; MacDonald et al., 2009) may explain significant proportions of age-related variance in executive functioning, episodic memory and processing speed (Bäckman et al., 2010). However, as the whole body is aging, it is not surprising that age-related changes in the autonomic nervous system (ANS) have also been implicated in cognitive aging (Parashar et al., 2016; Baker et al., 2018). Specifically, age-related changes in the ANS and particularly the balance between the sympathetic nervous system (SNS) and the parasympathetic nervous systems (PNS) that affect the pupils in the eyes and all other non-central nervous systems (CNS) organs including the heart, lungs, gut and mucous membrane (Pfeifer et al., 1983; McLean and Le Couteur, 2004; Shimazu et al., 2005; Parashar et al., 2016; Strickland et al., 2019) and interact closely with stress hormones (i.e., cortisol) of the hypothalamus-pituitary-adrenal (HPA axis; Gupta and Morley, 2011; Gaffey et al., 2016) have also been implicated. Interestingly, age-related changes in the functioning of the SNS have only recently begun to be discussed in relation to explaining cognitive decline (Beer et al., 2017; Knight et al., 2019; Dalise et al., 2020).

Neurocomputational work on the triad between aging, cognition, and DA has suggested that reduced DA activity increases neuronal noise (i.e., random electrical fluctuations generated within neuronal networks that are not associated with encoding a response to stimuli; Li et al., 2001). Increases in neuronal noise have been suggested to have various functional consequences, including less distinctive neuronal representations of perceptual stimuli, increased interference between different functional networks (Li and Sikström, 2002) and impaired interactions between intrinsic neuronal and perceptual noise which may collectively result in impaired performance on cognitive tasks (Li et al., 2006; Bäckman et al., 2010). This suggestion is in line with several other sensory theories of aging and cognitive decline i.e., the *Information Degradation Hypothesis* described above (Schneider and Pichora-Fuller, 2000). Despite the commonly reported role of the dopaminergic system in cognitive performance (Li and Sikström, 2002; Aalto et al., 2005; Bäckman et al., 2010), the implication that it is the *cause* of cognitive decline seen across the lifespan must be interpreted with caution. Moreover, the observations of altered DA levels during cognitive-behavioral tasks have been scrutinized in a systematic review by Egerton et al. (2009), who noted that uncontrolled head movements during behavioral tasks may ultimately confound the conclusions relating to regional cerebral blood flow changes during task performance. Furthermore, although DA is often considered as the neuromodulator affecting noise signals in neural systems (Li and Lindenberger, 1999; Erixon-Lindroth et al., 2005) there are many other monoamines such as noradrenaline and serotonin that modulate neural network activation (Jacob and Nienborg, 2018) and must eventually be researched before an entirely comprehensive model of cognitive aging can be achieved.

### The Association Between Perceptual Abilities and Decline in Cognitive Performance

The association between perceptual abilities and cognitive performance was demonstrated in a study conducted by Lindenberger and Baltes (1994) who reported that sensory variables such as visual acuity, auditory acuity, as well as balance-gait, predicted 59% of the total variance in general intelligence. Similarly, a study conducted by Humes et al. (2013) indicated that the relationship between age and cognitive performance (measured with WAIS subsets) was mediated by sensory function when the factor was based on a composite measure of auditory, visual and tactile perception. On the other hand, a study by Füllgrabe et al. (2015) failed to find any age group differences in the auditory forward and backward digit span tasks in a sample of healthy young and older participants who were audiometrically matched, highlighting the correlation between reduced speech intelligibility and performance on auditory based cognitive measures. Most recently, age-related physiological and behavioral changes in the visual system have been demonstrated using both flicker fusion thresholds as a behavioral measure of the function of the fastest conducting Magnocellular (M) pathway and multifocal Visually Evoked Potentials (mfVEPs) as a measure of retino-cortical latency of the two major M and Parvocellular (P) subcortical visual pathways. Multifocal VEPs showed increases in latency with the age of both the M and P pathways, though the M generated peak latency increases were greater than those associated with the slower P pathways (Brown et al., 2019).

Despite the association between perceptual abilities and cognition being well-documented, some inconsistent findings remain (see Gennis et al., 1991; Hofer et al., 2003). In a review conducted by Roberts and Allen (2016), the authors strongly recommended assessing perceptual abilities beyond simple measures of visual and auditory acuity to corroborate the link between perception and cognition. Specifically, the use of more complex measures of suprathreshold temporal perceptual processing was recommended when investigating the cognitive decline in healthy aging (Roberts and Allen, 2016).

It is important to note that most of the literature assessing perceptual abilities and cognitive aging tend to focus on visual and auditory processing and neglect other sensory systems such as olfaction and somatosensory processing. Indeed, olfactory dysfunction is reported in >50% of older adults aged over 65 years and has been associated with impairments in cognitive abilities such as memory decline (see Attems et al., 2015 for a review). Similarly, the somatosensory system is reported to decline in healthy aging (Heft and Robinson, 2017; Strömmer et al., 2017) though somatosensory processing and its association with cognitive aging has not been extensively studied. Though generalized explanations specific to sensory integrity as described above have been influential in the aging literature, they also neglect other biological factors of aging which are related to cognition, including vascular hypertension-related changes (Liu et al., 2018). Furthermore, few studies have considered the contribution or confounds of long term blood pressure medication or the increasing evidence that cerebral small blood vessel disease (CSVD) is emerging as a principal risk factor for cognitive impairment in apparently healthy adults (Liu et al., 2018; Jiménez-Balado et al., 2019).

## The Processing Speed Theory

Theories postulating that processing speed underlies the observed decline in complex cognitive abilities were pioneered as early as the 1960s by Birren (1965) who observed that processing rate for a broad range of cognitive tasks increased as a function of older age. This theory was validated and expanded through an extensive body of work conducted by Salthouse (1985, 1996), who postulated the influential theory of cognitive aging, namely, the *Processing Speed Theory of Adult Age Differences in Cognition* (Salthouse, 1996). This theory suggests that a slowing in the speed at which cognitive processes can be executed, underlies the decline observed in more complex cognitive functions, including memory, problem-solving and reasoning (Salthouse, 1996). Specifically, Salthouse (1996) proposed that older adults have impairments in two interconnected mechanisms relating to processing speed, i.e., a *limited time mechanism*, and a *simultaneity mechanism*, which together underlie the deficits observed in higher-order cognitive abilities. The *limited-time mechanism* suggests that older adults require more time to process early operations of a cognitive task than do younger individuals and consequently greatly restrict performance on later operations, as a large portion of available time is already occupied. The *simultaneity mechanism* is based on the notion that products of early processing may no longer be available i.e., information is forgotten by the time later processing is complete (Salthouse, 1996). This theory has been influential in the cognitive aging literature, and its fundamental underpinnings remain relevant to modern-day experimental research, particularly as a means to explain age-group differences in cognitive performance (Salthouse, 2000; Costello et al., 2010; Eckert, 2011; Ebaid et al., 2017; Ebaid and Crewther, 2018, 2019; Brown et al., 2019). A major limitation of this body of work is the predominant use of paper-pencil tasks with aged populations, which inextricably introduces the confounding element of motor speed (Ebaid et al., 2017). This is even more prominent in populations with neurological impairments, i.e., following a stroke, which significantly impairs motor ability (Langhorne et al., 2009). For example, the Digit Symbol Substitution test from the WAIS-R (Wechsler and De Lemos, 1981) was one of the measures utilized in Salthouse (1992) to inform the premise of his *Processing Speed Theory* (Salthouse, 1996). Though psychomotor speed as a factor contributing to scores was acknowledged in early research by Salthouse, no data has been provided where motor speed was statistically covaried. Instead, the premise of the argument has assumed that even though motor speed may be a factor influencing paper-pencil performance on cognitive tasks, such tasks were adequate measures and were not substantially confounded by motor processes (Salthouse, 1992, 1996). Since then, several other studies based on findings from paper-pencil measures from the Processing Speed Index (PSI) of the WAIS without explicit consideration of hand-motor speed confounding results, have supported findings in line with the *Processing Speed Theory* (i.e., Joy et al., 2000; MacDonald et al., 2003; Lu et al., 2011). Furthermore, although the *Processing Speed Theory* has considerable supporting evidence in explaining the age-related decline in cognitive performance, it is important to acknowledge that the assumptions provided are broad, particularly when inferring cognitive skills from motor reaction time measures given that they are reliant on general sets of cognitive (usually visual) and motor skills. Considering this, tests that rely on a manual response are less able to dissociate specific cognitive processes such as selective and temporal attention and perceptual rate of processing without being confounded by motor performance.

Alternative non-motor assessment of cognitive processing speed can be achieved *via* psychophysical Inspection Time (IT) tasks which measure early cortical perceptual speed by estimating threshold exposure duration required to successfully discriminate and identify a familiar visual stimulus (Vickers, 1970; Wilson et al., 1992). Similarly, a Change Detection (CD) task (Becker et al., 2000; Rutkowski et al., 2003) without a motor component, can also provide useful data regarding exposure time needed to complete an IT array task, embedding of the array in short term memory plus an additional working memory task requiring a decision as to whether a second visual array is the same or different to first presented. Indeed, such psychophysical IT and CD tasks can serve as effective tools for measurement of information/cognitive processing time without the confounding factor of motor slowing which often accompanies paper-pencil measures of speed (Ebaid et al., 2017; Ebaid and Crewther, 2018, 2019; Crewther and Ebaid, 2019).

## The Inhibitory Deficit Hypothesis

The *Inhibitory Deficit Hypothesis* (Hasher and Zacks, 1988) is an attention-based model of age-group differences in cognitive abilities. The premise of the *Inhibitory Deficit Hypothesis* is that good task performance requires efficient processing of relevant information while simultaneously inhibiting irrelevant information, and that any deficit in this inhibitory-regulation process will result in heightened distractibility and sustained access to irrelevant information, greater reliance on environmental cues, reduced working memory capacity, and poorer retrieval of task-relevant details (Hasher and Zacks, 1988). The *Inhibitory Deficit Hypothesis* suggests that age-related declines in cognitive tasks are a result of older adults being less efficient in maintaining selective attention to task-relevant stimuli while simultaneously inhibiting task-irrelevant information. As a result, goal-irrelevant information occupies the limited attention and working memory capacity, at the expense of relevant information, thus resulting in worsened task performance by older adults (Hasher and Zacks, 1988). Though this argument has strong theoretical merit and has been supported in several studies (i.e., Kramer et al., 1994; Andrés et al., 2008; Verhaeghen, 2011), an age-related inhibition deficit has not been consistently reported (Ludwig et al., 2010; Rey-Mermet and Gade, 2018).

Several suggestions have been put forward to account for the discrepancy in results. Such suggestions include the possibility that differences in cognitive functioning and cognitive reserve in the older adult sample varied from study to study and those with more preserved cognitive function attenuated the inhibition deficit (Rey-Mermet and Gade, 2018). It has also been suggested that task-to-task discrepancy which supposedly measure inhibitory control, as well as the differences in methods used by researchers to assess and statistically account for processing speed, elucidate different results across studies (see Rey-Mermet and Gade, 2018 for a recent meta-analysis on this issue). Furthermore, tasks used to measure inhibitory control often rely on accuracy and response speed on a computerized task, or verbal reaction times i.e., Stroop-tasks (Stroop, 1935), which again introduce the potential confound of hand motor speed or slower orofacial movement in an aged population. A less commonly used measure is eye-tracking, despite its known usefulness and robustness in measuring cognitive domains such as attention and inhibition (Harkin et al., 2012; Crewther and Ebaid, 2019). More specifically, oculomotor functions including visual fixations and saccade duration are surrogate measures of visual attention shifting (Mohler et al., 1973; Wurtz and Goldberg, 1989) and are known to be affected by eye movements and rate of conduction of major retino-cortical pathways i.e., the M pathway from stimulus onset to arrival in the cortex (Brown et al., 2019).

## The Scaffolding Theory of Aging and Cognition (STAC)

The Scaffolding Theory of Aging and Cognition (STAC; Park and Reuter-Lorenz, 2009) integrates evidence from structural and functional neuroimaging to provide a conceptual model of cognitive aging. The model proposes that the level of cognitive functioning that an older individual achieves, is a consequence of both neural/functional deterioration as well as “compensatory scaffolding” which is utilized to attenuate the adverse effects of the neural and functional decline. The neural and biological deterioration that occurs with normal aging has been recently shown to include latency of visual information conduction from retina to cortex (Brown et al., 2019) and has been suggested to include cortical thinning, regional atrophy, loss of white matter integrity, DA depletion, decreased memory-related recruitment of medial temporal lobe regions (Cabeza et al., 2004; Gutchess et al., 2005) and dysregulation of the default mode network (Park and Reuter-Lorenz, 2009). Authors describe compensatory scaffolding as a “positive” or adaptive form of plasticity that enables older adults to engage supplementary neural circuits that provide the additional computational support required to preserve cognitive function in the face of neurofunctional decline. For example, the theory postulates that increased frontal activation exhibited by healthy older adults when working on the same cognitive task as younger adults is a marker of an adaptive brain which engages in “scaffolding” in response to declines posed by deficits in the efficiency of neural structures (Park and Reuter-Lorenz, 2009).

Despite such suggestions, it is unclear exactly how compensatory or scaffolding mechanisms are utilized, and how much these compensatory pathways contribute to “better” performance. According to the theory, the scaffolding mechanisms are suggested to be protective in the aging brain, and the ability for older persons to use such mechanisms are reportedly strengthened by factors including higher levels of education, physical activity, and exercise (Erickson and Kramer, 2009), while depression is reported to impair scaffolding abilities (Tsai, 2003). However, from the viewpoint of the STAC, it is still unclear as to how such lifestyle factors strengthen the “scaffolding” mechanisms. If lifestyle factors had this type of positive impact, it can be assumed that increased level of education and physical activity may *protect* against cognitive decline using cognitive reserve and healthy vascular systems with minimal associated neuroinflammation, however, mixed results still exist within this realm (Stern, 2002; discussed in more detail below).

## The Cognitive Reserve Hypothesis

The *Cognitive Reserve Hypothesis* postulates that individuals who possess a greater ability to recruit and utilize particular brain regions are better able to cope with a greater level of age-related brain pathology i.e., that associated with mild cognitive impairment (MCI) before a clinical diagnosis is reached (Stern, 2002). It has also been suggested that this is enabled using neural compensation and recruitment of additional brain regions that lead to unimpaired behavioral performance (Stern, 2002). This notion stems from the observation of individuals who were functioning below levels of clinical impairment, despite brain pathology that other individuals who demonstrated clinical impairments also showed (Davis et al., 1999; Riley et al., 2002). Methods of quantifying “cognitive reserve” typically involve inferring *reserve* from proxy measures such as educational attainment or current occupation (Jones et al., 2011) which are suggested to “*supply reserve in the form of a set of skills or repertoires that allows some people to cope with pathology better than others*” (Scarmeas and Stern, 2003, p. 625). Indeed, several studies have supported the notion that greater educational attainment can slow the trajectory of age-related cognitive decline and can promote the capacity to process tasks more efficiently (Bennett et al., 2003; Cabeza et al., 2016). Additional factors contributing to cognitive reserve are also reported to include complex occupation attainment (i.e., professional occupations or managerial positions), physical and leisure activity (Darwish et al., 2018), as well as dietary patterns (Bowman and Scarmeas, 2019). Though this theory has been influential in providing a framework for intact cognitive function in the face of pathology, the body of research is based on proxy measures of “cognitive reserve” which vary from study to study. More specifically, “cognitive reserve” is not merely represented using one accepted measure across the scientific literature. Rather, the measures chosen to represent “cognitive reserve” in the literature, depends on the researchers’ theoretical concept of what *reserve* means (Stern, 2002). Anatomical measures including brain size, head circumference, dendritic branching, and synaptic count have been suggested as effective measures of reserve (Stern, 2002), though are seldom utilized in cognitive aging research. Although the *Cognitive Reserve Hypothesis* has provided insight into cognitive aging, caution must be taken when interpreting such findings.

## The Frontal Aging Hypothesis

The *Frontal Aging Hypothesis* (Jackson, 1958; Dempster, 1992) is based on the notion that selective frontal lobe pathology in the form of reduced volume, metabolism and a simultaneous decline in gray matter (Raz et al., 2005) and white matter integrity (Barrick et al., 2010) underlie the cognitive deficits observed in healthy aging, which has mainly been demonstrated using functional magnetic resonance imaging (fMRI) or positron emission tomography (PET; Jackson, 1958; Dempster, 1992; West, 2000). This notion is also fundamental to the view that age-related “involution” initiates in the frontal lobe, in turn leading to cognitive decline in apparently healthy aging (Dempster, 1992). The basis of this theory is still relied upon in recent aging literature (i.e., Calso et al., 2019), with some research even concluding that *“the prefrontal cortex leads most, if not all, other areas in the aging process”* (Dempster, 1992, p. 51). In line with the *Frontal Aging Hypothesis*, research suggests that unlike anterior cortical regions, posterior brain regions are spared through the aging process (Hartley, 1993). The theory that frontal areas of the brain are particularly vulnerable to insult has even extended to theories into the origins of Alzheimer’s disease (AD; Rapoport, 1990). However, criticisms to this theory were pioneered as early as the year 2000 by Greenwood (2000) who provided a thorough critical review of the *Frontal Aging Hypothesis* and highlighted that healthy older adults are also impaired in cognitive abilities that are largely independent of prefrontal areas, such as visuospatial attention, face recognition, and repetition priming. Furthermore, most recent literature particularly that associated with whole-brain imaging demonstrates that additional cortical areas including the temporal and parietal lobes also show compromised integrity with age (McGinnis et al., 2011; Farina et al., 2017; Fukuda et al., 2018; Klöppel et al., 2018; Grassi et al., 2019). Such findings argue against localizing general cognitive decline to the frontal lobe, given that most cognitive functions rely on networks of regions (Greenwood, 2000; Farina et al., 2017; Fukuda et al., 2018; Klöppel et al., 2018; Grassi et al., 2019). Indeed, it has since been suggested that a network-based approach is a better way to conceptualize cognition and cognitive aging, as this approach also emphasizes the role of broader neural networks without minimizing the role of the frontal lobes (see Greenwood, 2000). A substantial body of work in this area has been conducted by Mesulam (1990, 1994, 2009).

## The Hemispheric Asymmetry Reduction in Older Age (HAROLD) Model

The Hemispheric Asymmetry Reduction in Older Age (HAROLD) model (Cabeza, 2002) is based on the premise that age is related to decreases in lateralization of brain function, which stem from fMRI observations that young and older adults recruit different neural networks during the same cognitive task (particularly episodic and working memory tasks). Specifically, it has been reported that young adults display left Prefrontal Cortex (PFC) activation during verbal working memory tasks and right PFC activation during spatial working memory tasks (Reuter-Lorenz et al., 2000), whereas older adults demonstrate bilateral activation of the PFC while engaging in both verbal and spatial working memory tasks (Reuter-Lorenz et al., 2000). The age-related decreases in lateralization proposed by this model are suggested to occur due to neural changes and a global reorganization of neurocognitive networks which result in bilateral activation during cognitive tasks, which may be reflective of compensatory processes (Cabeza, 2002). It is important to note that the HAROLD model has been exclusively described for the PFC, and insight into this model has come from studies predominantly using episodic and working memory tasks (for a review see Berlingeri et al., 2013). Again, such explanations are limited to specific brain regions and neglect the more commonly accepted neural network-based perspective of the human brain.

## The Compensation-Related Utilization of Neural Circuits Hypothesis (CRUNCH)

The CRUNCH model is based on the concept of neural compensation and attempts to explain the distinctiveness of neural representations between young and older adults while performing the same cognitive task (Reuter-Lorenz and Cappell, 2008). Specifically, the CRUNCH model suggests that declines in neural efficiency across the lifespan results in older adults recruiting more neural resources predominantly in the dorsolateral PFC compared to young adults when task demands are low (Reuter-Lorenz and Cappell, 2008). However, as task demands increase, neural activation for younger adults exceeds that of older adults, and task performance for older adults is also impaired. Put simply, when task demands exceed a certain level of difficulty, the aging-brain “falls short” of sufficient activation levels, and task performance declines compared to the younger adults, and this trade-off underpins the premise of the CRUNCH model (Reuter-Lorenz and Cappell, 2008). This may be due to older adults’ neural resources being limited by the bioenergetics of the mitochondria of neurons that are known to decrease in number with age (see Haas, 2019). Task demands have also recently been shown to alter time perception of task duration, whereby the duration of a low-cognitive demand task is perceived as substantially shorter than objective time in healthy young and older adults (Ebaid and Crewther, 2018).

Models such as the STAC, HAROLD and the CRUNCH are based on neural compensation in one way or another, and postulate that older adults can perform as well as young adults on cognitive tasks depending on the capacity to recruit additional neural networks, which are often indicated by increased effort i.e., increases in energy/neural activation. Another important consideration which opposes the notion that increased activation is indicative of an adaptive brain stems from the *Neural Efficiency Hypothesis* (Haier et al., 1988) which postulates that more efficient brain functioning is indicated by *lower* brain activation compared to less intelligent individuals while working on the same cognitive task (predominantly memory tasks). This phenomenon was first described by Haier et al. (1988) using PET. More specifically, Haier et al. (1988) demonstrated that healthy young adults with a lower non-verbal IQ score on the Ravens Advanced Progressive Matrices (RAPM) showed more cortical activity throughout the brain when working on the same cognitive task, compared to those individuals with higher RAPM scores. However, this hypothesis was based on a relatively small sample of 30 right-handed healthy young male volunteers and has not been explicitly translated to the healthy aging literature.

## The Cardiovascular System and Age

### Hypertension

Cardiovascular system integrity decreases with age (Xu et al., 2017), with Western lifestyle risk factors such as overeating, obesity, and lack of exercise contributing to worse cardiovascular health (Casas et al., 2018) including hypertension (Singh et al., 2017; Trudel et al., 2019). In Australia, more than 40% of adults aged 65 and above have uncontrolled hypertension, with prevalence rates as high as 51% for adults above 85 years (Australian Institute of Health and Welfare, 2019). Hypertension is considered a worldwide epidemic and affects ~1.13 billion adults globally (World Health Organisation, 2019). Indeed, hypertension is a risk factor for CSVD and neuroinflammation (Allison and Ditor, 2014; Chen et al., 2016), and in turn, cognitive decline. The link between characteristics of CSVD and cognitive decline has been reviewed in a recent study conducted by Liu et al. (2018) who explored hypertensive vasculopathy factors including small vascular lesions, inflammatory reactions, hypoperfusion, and blood-brain barrier damage. They found that all factors associated with hypertension are vital prognostic indicators of the development of cognitive impairment, particularly when blood pressure management is poor (Liu et al., 2018). As alluded to earlier in this review, ANS dysregulation has been suggested as a pathophysiological link between hypertension and negative affect, particularly anxiety (Bajkó et al., 2012). In another study conducted by Jiménez-Balado et al. (2019), authors investigated how changes in CSVD lesions over 4 years relate to cognitive decline and incident MCI in 345 hypertensive patients (median age = 65). Jiménez-Balado et al. (2019) demonstrated that patients with marked progression of periventricular white matter hyperintensities showed a significant decrease in global cognition (as measured by the Dementia Rating Scale—second version). Jiménez-Balado et al. (2019) also reported that patients with marked progression of periventricular white matter hyperintensities had a higher risk of MCI (i.e., subjective cognitive impairments below clinical thresholds) compared to those without progression. Also, a longitudinal study examining the association between cognitive dysfunction, hypertension and cognitive deterioration over 5 years in 990 subjects (mean age of 83 years) with cognitive impairment but no diagnosis of dementia, demonstrated that among the individuals with executive function deficits but no memory impairments, 57.7% of subjects with hypertension progressed to dementia compared with only 28.0% with normotension (Oveisgharan and Hachinski, 2010). It was concluded that hypertension predicts progression to dementia in older subjects, and control of hypertension could prevent progression to dementia in one-third of subjects with cognitive impairment (Oveisgharan and Hachinski, 2010).

In another study conducted by Jiménez-Balado et al. (2019), authors investigated how changes in CSVD lesions over 4 years relate to cognitive decline and incident MCI in 345 hypertensive patients (median age = 65). Jiménez-Balado et al. (2019) demonstrated that patients with marked progression of periventricular white matter hyperintensities showed a significant decrease in global cognition (as measured by the Dementia Rating Scale—second version).

The relationship between hypertension and cognitive impairments is not always consistently reported however, with some studies that have examined hypertensive vasculopathies such as cerebral microbleeds and cognitive abilities reporting no difference in performance based on the presence of vascular pathologies (Rabelo et al., 2017). Specifically, Rabelo et al. (2017) assessed cognitive performance using neuropsychological measures including the Mini-Mental State Exam (MMSE), the Rey Auditory Verbal Learning Test (RAVLT) and the auditory forward and backward digit span in a sample of patients with AD, MCI, and cognitively healthy adults. The results showed no association between cerebral microbleeds and cognitive performance, and no significant differences in cognitive performance when considering the presence of cerebral microbleeds (Rabelo et al., 2017). It may be the case that some markers of hypertensive vasculopathies are not universally effective tools as biomarkers for AD and MCI, particularly in the early phases (Liu et al., 2018).

As 20% of cardiac output of the human body is devoted to meeting the brains energy demands (Attwell et al., 2010), protection from hypofusion and ischemic damage is vital to enable cerebral blood flow during fluctuations in arterial pressure (Jackman and Iadecola, 2015). This process is significantly and chronically impaired by CSVD and consequently takes a large toll on the individual as well as the health care system (for a recent review see De Silva and Miller, 2016). Indeed, vascular integrity and cognition in aging are gaining increased attention in the literature and making such issues necessary for inclusion in future considerations in work on explanations of cognitive aging (Gąsecki et al., 2013; Wang et al., 2015; De Silva and Miller, 2016).

### Obesity

The prevalence rates for being overweight or obese in adults aged 65–74 years in Australia is ~80% for males and ~69% for females (Australian Institute of Health and Welfare, 2018a). Obesity has also been associated with more rapid cognitive aging (Anstey et al., 2011; Dahl and Hassing, 2013). In a meta-analysis conducted by Anstey et al. (2011), the authors reported that obesity, underweight and overweight Body Mass Index (BMI), are all associated with an increased risk of dementia compared to normal-weight individuals. Furthermore, in a systematic review conducted by Dahl and Hassing (2013), it was concluded that midlife obesity had detrimental consequences on cognitive processing later in life. More specifically Dahl et al. (2010) showed that early midlife (*M*_age_ = 42 years) obesity indicated by BMI was associated with a steeper decline in general cognitive ability as measured with a neuropsychological test battery 21 years after the initial assessment, even after controlling for age, sex, education, cardiometabolic factors, alcohol use, smoking, twinness, and cohort. Dahl et al. (2013) also investigated the association between BMI measured twice across midlife (*M*_age_ = 40 and 61 years, respectively) and cognitive changes measured by WAIS subtests. Dahl et al. (2013) found a significant relationship between midlife obesity and a decline in perceptual speed, spatial abilities, and verbal abilities, with a steeper decline in verbal and spatial abilities. Such findings emphasize the need to consider factors such as body weight when assessing cognitive performance in aged populations.

### Hyperlipidaemia

Prevalence rates of hyperlipidemia for adults in Australia aged 65–64 years are reported to be as high as 78% for men and 84% for women (Australian Institute of Health and Welfare, 2018b). Hyperlipidemia has also been identified as a potential risk factor for cognitive decline in late life (Cheng et al., 2014). Indeed, a study conducted by Cheng et al. (2014) reported a significant relationship between cholesterol levels and cognitive function in 1,889 participants (*M*_age_ = 73.45 years) which was dependent upon homocysteine levels. More specifically, Cheng et al. (2014) measured serum total cholesterol, high-density lipoprotein, triglycerides, and homocysteine levels in fasting blood samples, and used a composite cognitive score comprising of nine tests such as the Community Screening Instrument for Dementia, the ten-word list learning and word list recall test (Morris et al., 1989), and the Animal Fluency Test (Isaacs and Akhtar, 1972). Results revealed an inverse U-shaped relationship between total cholesterol level and cognitive score, indicating that both low and high cholesterol levels were associated with worse cognitive performance (Cheng et al., 2014). Results also revealed that in participants with high homocysteine levels, no significant association between cholesterol and cognition was found, which suggests an interactive role between cholesterol and homocysteine on cognitive function in elderly populations (Cheng et al., 2014). Despite the influence of factors such as obesity and hypolipidemia on cognitive aging, they have not been considered or implemented in a single cognitive aging theory to date. As such, a multifactorial understanding of cognitive processing in late life is unlikely, when applying current theories of aging to experimental aging research in populations where the prevalence of such conditions is high, i.e., in Australia (Samper-Ternent and Al Snih, 2012).

## Anxiety and Depression in Aging

Chronic negative affect, i.e., depression and anxiety are often associated with aging (Bryant et al., 2008; Perna et al., 2016; Burhanullah et al., 2020). However, it must be emphasized that acute anxiety is an innate biologically adaptive response to potential environmental threats mediated by the HPA axis that affects human behavior and cognition (Robinson et al., 2013). On the other hand, prolonged anxiety and over-activation of the HPA-axis can also be maladaptive, leading to distortions to the stress response (Vashist and Schneider, 2014; Herman et al., 2016; Strickland et al., 2019). Thus, prolonged anxiety has been proposed as a causal factor influencing the role of neuropathologic processes and leading to cognitive decline and dementia (Gulpers et al., 2016). Unlike depression which has received substantial attention in the cognitive aging literature concerning its link with MCI and dementia (Diniz et al., 2013), the effects of state and trait anxiety are still unclear (Beaudreau and O’Hara, 2008). Specifically, late-life depression has been associated with cognitive impairments in domains including processing speed, language processing, episodic memory, visuospatial skills, verbal fluency, and psychomotor speed (Morimoto et al., 2014), as measured by neuropsychological test batteries (Sheline et al., 2006). Such declines have been attributed to subcortical structures such as the hippocampus (Hickie et al., 2005; Xie et al., 2013) whereby magnetic resonance imaging (MRI) studies have demonstrated that individuals with major depressive disorders have reduced whole-brain and left and right hippocampal volume (Hickie et al., 2005). In addition to this, other studies have also suggested that hippocampal size and function are diminished proportionately to the duration of prior hypercortisolemia (Lupien et al., 1998), as well as in patients with elevated glucocorticoids (Peavy et al., 2007). Based on such findings, it has been suggested that in the context of chronically elevated glucocorticoids such as in hypercortisolemia associated with major depression, glucocorticoids contribute to hippocampal cell injury and death, and in turn, impair cognitive functions including spatial memory (Lupien et al., 1998) and autobiographical memory (Buss et al., 2004). The association between cortisol levels and poor cognitive function was reviewed in a recent study conducted by Ouanes and Popp (2019) who found that elevated cortisol level was associated with poorer processing speed, episodic and spatial memory, and language. There is also evidence to suggest an interaction between hypercortisolism and brain-derived neurotrophic factor (BDNF), in that HPA-axis activation increases the glucocorticoid level, which in turn decreases BDNF expression in the hippocampus (Hansson et al., 2003). Furthermore, it has been reported that the glucocorticoid receptor interacts with the specific BDNF receptor (TrkB), and elevated glucocorticoid interferes with BDNF signaling. Indeed, such alterations in BDNF signaling has been suggested to play a role in the structural changes in individuals with depression (Kunugi et al., 2010).

Though late-life anxiety and cognitive aging are less investigated compared to depression, what is known are the biological characteristics of chronic anxiety, which in turn may negatively impact cognitive performance (see Gulpers et al., 2016 and Robinson et al., 2013 for a recent systematic review and meta-analysis).

Gulpers et al. (2016) proposed several potential hypotheses for anxiety leading to cognitive decline, one of which included hypercortisolism, i.e., higher levels of cortisol which in turn negatively affects performance on cognitive tests (Rosnick et al., 2013). This effect has been suggested to result from overstimulation of glucocorticoid receptors in the medial temporal lobe which results in hippocampal atrophy (Erickson et al., 2003). Increased cortisol has also been associated with smaller total brain volume, particularly in gray matter regions (Geerlings et al., 2015). This association was demonstrated in a large sample of 4,244 individuals without dementia (*M*_age_ 76 ± 5 years, 58% women). For a recent review on hypercortisolism and its effects on the brain see Ouanes and Popp (2019). Furthermore, a role for inflammation i.e., increased levels of cytokines including interleukin-6 and tumor necrosis factor (TNF), has also been suggested as a possible causal pathway between anxiety and cognitive impairment (Reichenberg et al., 2001; Gulpers et al., 2016). Indeed, previous research conducted by Menza et al. (2010) have reported that increased levels of inflammatory cytokines are significantly correlated to worse cognitive performance, where cognitive performance was examined using a composite score comprised of raw scores on tests including the MMSE, the forward and backward digit span tests, and Stroop color-word tests (Stroop, 1935). Gulpers et al. (2016) have also suggested that decreased levels of BDNF associated with anxiety may also explain the relationship between anxiety and cognitive impairment, given BDNF’s essential role in regulating cellular processes that underlie cognition (Lu et al., 2014). The prevalence of anxiety in older adults aged over 60 years can range from 15% in community samples and 56% in clinical samples i.e., hospitalized populations (see for a review, Bryant et al., 2008). Despite this, anxiety as a factor contributing to task performance is seldom explicitly addressed in theories of cognitive aging. Thus, cognitive aging research may benefit from utilizing an anxiety screening instrument such as (but not limited to) the Depression Anxiety Stress Scale (DASS-21; Lovibond and Lovibond, 1995), the Generalized Anxiety Disorder Questionnaire (GADQ-IV; Newman et al., 2002), the Hamilton Anxiety Rating Scale (HARS; Hamilton, 1959), or the Hospital Anxiety and Depression Scale (HADS; Zigmond and Snaith, 1983).

## Limitations

Though this review has attempted to critically evaluate current prominent theories of cognitive decline with age while emphasizing the need for a newer more comprehensive systems neuroscience approach to cognitive aging, some limitations need to be acknowledged and highlighted. First, it is important to note that the authors selected common prominent cognitive aging theories that are primarily based on or derived from cognitive psychology frameworks. The authors willingly acknowledge that many other theories exist in the literature that attempt to explain the decline in cognitive domains with age. For example, the *Theory of Fluid and Crystallized Intelligence* (Cattell, 1963), which proposes that areas of cognition that comprise Fluid Intelligence (i.e., reasoning and problem solving) are more susceptible to age-related decline, whereas cognitive skills involved in Crystallized Intelligence which encompass knowledge acquired through previous learning, remain stable or improve with age, was not discussed in the current review. In addition, more biological theories such as *the hypothesis of metabolic reserve* (Stranahan and Mattson, 2015), which proposes that brains with more metabolic reserve are characterized by the presence of neuronal circuits that respond adaptively to perturbations in cellular and somatic energy metabolism, which in turn protect against cognitive decline, were not explicity considered in the current work. Furthermore, there are additional lifestyle factors that can contribute to cognitive decline in late life such as smoking (Anstey et al., 2007) and caloric intake (Geda et al., 2013) which were not critically discussed in this review. Therefore, some factors discussed in this review only apply to a subset of individuals, and not to all older adults who do not suffer from the health conditions previously discussed.

## Conclusions

**Figure 2:**
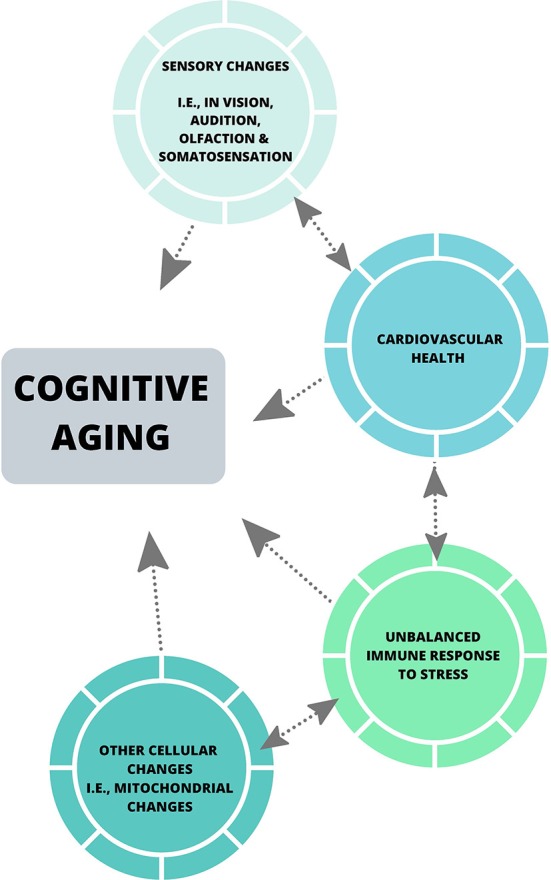
A systems biological viewpoint of cognitive aging.

Collectively, the theories discussed in this review have been influential in the cognitive aging literature, and have emphasized the importance of considering factors including sensory abilities in all aged populations when assessing cognitive performance, and the effect of motor speed on cognitive processing speed when utilizing paper-pencil measures of cognition. Some of these theories have also highlighted the impact of individual factors including level of education, occupational attainment (Darwish et al., 2018), dietary patterns (Bowman and Scarmeas, 2019) and physical activity levels, which may play a role in cognitive reserve (Stern, 2002). Despite this, much of the neuropsychological literature does not account for such factors, nor does it assess perceptual ability beyond the basic level of acuity (Füllgrabe et al., 2015; Roberts and Allen, 2016), and the most common measures of cognitive processing speed are still reliant on motor speed (see Ebaid et al., 2017). With the advances in neuroscientific tools [i.e., psychophysical measures of perceptual threshold and cognitive processing (Vickers et al., 1972), and eye-tracking] future theories of cognitive aging should aim to employ robust and diverse measures, such that a more accurate and holistic understanding of cognitive processing is encapsulated. Also, biological changes that commonly occur in later life including hypertension, hypercortisolism associated with anxiety, hyperlipemia, and obesity, have the potential to provide in-depth insights into cognition across the lifespan (see Figure 2). Most importantly, the implementation of a systems neuroscience approach to such global issues could enhance understanding of cognitive aging.

## Author Contributions

DE and SC contributed to the intellectual content of the manuscript and both contributed to drafting the manuscript.

## Conflict of Interest

The authors declare that the research was conducted in the absence of any commercial or financial relationships that could be construed as a potential conflict of interest.
